# Supplementary material for: Healthcare providers’ knowledge, attitudes, and perceptions from using targeted sequencing to diagnose and manage drug-resistant tuberculosis (DR-TB) in Eswatini
Source: PLOS Glob Public Health. 2025 Jun 12;5(6):e0004718. doi: 10.1371/journal.pgph.0004718 (PMC12161584; doi:10.1371/journal.pgph.0004718)
Supplement: S2 Checklist — (DOCX) [file pgph.0004718.s002.docx]

Inclusivity in global research

PLOS’ policy on inclusivity in global research aims to improve transparency in the reporting of research performed outside of researchers’ own country or community and ensures that PLOS publications reporting global research adhere to high standards for research ethics and authorship. Authors of relevant research articles may be asked to complete the questionnaire below, which outlines ethical, cultural, and scientific considerations specific to inclusivity in global research. This questionnaire may be requested when researchers have travelled to a different country to conduct research, if research uses samples collected in another country, research with Indigenous populations or their lands, or if research is on cultural artefacts. Researchers travelling to another country solely to use laboratory equipment will not normally be required to complete the questionnaire. However, the questionnaire can be requested at the journal’s discretion for any submission – if you have been requested to complete this questionnaire by the PLOS journal you submitted to, please do so.

Please complete the questionnaire below and include this as a Supporting Information file with your manuscript. Note that if your paper is accepted for publication, this checklist will be published with your article in the supporting information files. Please ensure that you reference the checklist in the main body of your manuscript. We suggest adding a subsection ‘Inclusivity in global research’ to your Methods section and adding the following sentence: “Additional information regarding the ethical, cultural, and scientific considerations specific to inclusivity in global research is included in the Supporting Information (SX Checklist)”

The questions have been designed to be applicable to a wide range of study types, and there are subsections for both human subjects research and non-human subjects research. If any of the questions are not relevant to your research please mark them as “N/A” as appropriate.

**Ethical considerations, permits and authorship**

*This section is applicable to all research types.*

Provide details as to who granted permissions and/or consent for the study to take place in the Methods section of your manuscript. This should include the names of **all** ethics boards, governmental organizations, community leaders or other bodies that provided approval for the study. If individuals provided approval refer to these people by their role or title but do not list their name(s).

Reported on page number: 8, line 188

If there were any deviations from the study protocol after approval was obtained please provide details of these changes in the Methods section of your manuscript.
Did this study involve local collaborators that are residents of the country where the research was conducted or members of the community studied? If you do not have any authors from said communities, please provide an explanation for this below.

Reported on page number: N/A

Local researchers with clinical experience were involved in reviewing the interview questions, planning the sampling method, and connecting the interviewer with interviewees (NS and BB from Eswatini). Researchers with origins in other countries were also involved in the study design (LMIC: DV from Zimbabwe (Managed the DR-TB program in Eswatini for over a decade), HIC: MM and AK from the United States of America; MM and AK are based in Eswatini, AK has worked in collaboration with the NTCP in Eswatini since 2016). Approval and guidance from the NTCP were sought prior to beginning the study. Members of the NTCP originate from Eswatini and other LMICs. (Local: SN, SM; LMIC: ST). Most of the authors have cultural heritages from low- and middle-income countries. Several of the authors (MM, AK, AM, and TN) are researchers from a high-income country who have collaborated in Eswatini for up to 12 years.

The first author of the study (MM) was based in Eswatini but is from a high-income country. Researchers based in Eswatini with long-term partnerships with the NTCP are on the senior authorship team, including AK and DV, who managed the DR-TB program at the NTCP for the past decade. The contributions of all researchers from Baylor Children’s Foundation Eswatini, the National TB Control Program, and the National TB Reference Lab were recognized through authorship.

We included early career researchers (MM, BB & NS) within the authorship team. They led the study and assisted with purposive sampling. MM is from a high-income country and was based in Eswatini for a 10-month period that encompassed the study implementation, analysis, and manuscript preparation. NS and BB are from Eswatini. NS has been accepted to the US Fulbright Program and will be obtaining an MPH in the United States; her work on the research team helped to support her successful Fulbright application.

Everyone listed as an author should meet PLOS’ criteria for authorship and all individuals who meet these criteria should be included in the author byline, rather than the acknowledgements. For further information please see the journal’s Authorship Policy.

**Human subjects research (e.g. health research, medical research, cross-cultural psychology)**

Did you obtain written informed consent from a representative of the local community or region before the research took place? How did you establish who speaks for the community? Details of written informed consent obtained from study participants should be reported separately in the Methods section of your manuscript.

Written and informed consent was obtained from each participant in the study. The details of informed consent are included in the methods section, pg. 9, line 213. The consent form that was read to participants is included in supplementary material 3.

How did members of the local community provide input on the aims of the research investigation, its methodology, and its anticipated outcome(s)?

Approval and guidance from the NTCP were sought prior to beginning the study.

When engaging with the local community, how did you ensure that the informed consent documents and other materials could be understood by local stakeholders?

Consent forms were read by several team members and approved by the Eswatini National Human Health Research Review Board. Additionally, we translated the consent form into SiSwati, however, all healthcare workers who were interviewed spoke English and were interviewed in English.

Will the findings of the research be made available in an understandable format to stakeholders in the community where the study was conducted (e.g. via a presentation, summary report, copies of publications, etc.)? Please provide details of how this will be achieved.

The findings were shared with the NTCP and will be presented as part of a Technical Working Group. The NTCP will be able to use the findings to inform the implementation of TS in Eswatini.

**Non-human subjects research using specimens/ animals collected as part of the study, or those housed in archival collections. Examples include archaeology, paleontology, botany and zoology.**

Did the permission you obtained from a local authority to perform the study include an agreement on access to outputs and benefit sharing? This may include procedures to enable fair distribution of the benefits and resources arising from the research performed. Please include any details of Prior Informed Consent and Benefit Sharing Agreements obtained. These may be required by field-specific regulations, for example the Convention on Biological Diversity (CBD) and the associated Nagoya Protocol.

N/A. This study did not include non-human specimens.

If the material used in your study was imported, please A) provide the year it was imported and B) indicate whether permits were obtained to import/export the materials used, C) provide details of any permits obtained. If this information is not available, please indicate this.

N/A. Material was not imported.

If you used archival specimens, please state how the material used in your study was acquired by the institute it is held in and provide details of any permits obtained for the original excavations/ sample collection. If this information is not available, please indicate this.

N/A. Archival specimens were not used.

How was the potential cultural significance of the materials collected in your study to local communities considered in your research design? Were Indigenous peoples and/or local researchers and institutions involved with archaeological excavations / collection of specimens? If so, please provide a description of their involvement.

N/A. Materials were not collected.

If your manuscript includes photographs of human remains please indicate whether authors obtained permission from descendants or affiliated cultural communities to do so.

N/A. Photographs of remains were not taken.
