# Supplementary material for: Healthcare providers’ knowledge, attitudes, and perceptions from using targeted sequencing to diagnose and manage drug-resistant tuberculosis (DR-TB) in Eswatini
Source: PLOS Glob Public Health. 2025 Jun 12;5(6):e0004718. doi: 10.1371/journal.pgph.0004718 (PMC12161584; doi:10.1371/journal.pgph.0004718)
Supplement: S1 Text — (PDF) [file pgph.0004718.s003.pdf]

BAYLOR COLLEGE OF MEDICINE Children's Foundation ESWATINI	Feasibility, Usability and Acceptability of stool-based qPCR to diagnose TB in children aged up to 8 years and HIV- positive adults in Eswatini: a qualitative study	**Stool-4-TB-SBS**
	**SEMI-STRUCTURED INTERVIEW GUIDE**<br>(to assess tNGS interpretation from stool and sputum in Healthcare providers)	

Instructions

1. Explain to the participant that:

- This interview is part of a qualitative study, assessing the Feasibility, Usability, and Acceptability of stool-based tNGS to diagnose DR-TB in children aged up to 8 years and HIV- positive adults in Eswatini;
- Then, with this interview, we seek to explore:
    - (i) the healthcare provider's experiences in the TB care sector, and;
    - (ii) their perceptions and opinions regarding to feasibility, usability, and acceptability of tNGS on stool and sputum to diagnose DR-TB
- They were invited to interview because their contribution is very important to address the themes/topics above.
- The conversation will last between 30 to 60 min (1 hour), depending on the course of the interview.
- The interview be recorded if the interviewee allows it. Explain that we record and take notes to ensure that we can capture as much information as possible given by her/him. All recorded information will be confidential and he/she will not be identified by his/her real name. Also, explain that he/she is free to not accept the interview recording. In all cases (accepting to record or not), the interviewer will take notes during the conversation.

DEMOGRAPHIC INFORMATION

<table>
<tr><td>Healthcare Provider data</td><td colspan="2">Initials: ________
DR-TB clinic: ________________________________
Clinic geography: ____________________________
Occupation: _______________________________</td></tr>
<tr><td>Date and interview Location</td><td colspan="2">Date: ____________________________________
Location: _________________________________</td></tr>
<tr><td>Spoken Language</td><td colspan="2">Official Language:___________________________
Local Language:______________________________</td></tr>
<tr><td>Interview result</td><td>Recorded Not recorded Reason: ____
Completed Interrupted Reason: ___________________</td><td>Impossible to complete
To be completed on (date):</td></tr>
<tr><td colspan="3">Was read the informed consenting to the participant? Yes |_| No |_|</td></tr>
</table>

**Role in DR-TB care**<br>1. How would you describe your role in caring for patients with DR-TB<br>• What are your responsibilities?<br>• Are you engaged in research activities? explain.<br>2. How would you describe the demand for DR-TB healthcare services?<br>• Describe your patients' demographics— (ie. sex, age, HIV status, distance from clinic, socioeconomic status)<br>• What is the demand for your services — number of patients per day?	

tNGS for DR-TB diagnosis

1. Describe how you currently diagnose DR-TB
    1. What do you think works well?
    2. What do you think could be improved?

2. Describe what you know about using targeted next-generation sequencing for the diagnosis of DR-TB.
    1. What are the current guidelines for reporting DR-TB?

3. How has the use of sequencing for diagnosing DR-TB evolved in your clinic?

4. Describe the process of using tNGS to diagnose DR-TB.
    1. What steps do you take?/What is your role?
        1. What works best?
        2. What would you change?
    2. Does tNGS introduce more or less work for you? Explain.
    3. Describe your communication about tNGS and DR-TB with patients, the advisory committee, public health officials, and clinicians about tNGS.

5. Describe your experience interpreting results.

1. How do you interpret results when phenotypic and sequencing don’t agree?	

**Stool based tNGS for DR-TB diagnosis**<br><br>1. What do you think about using stool samples for diagnosing DR-TB?<br>2. How do you think this would affect the diagnosis of DR-TB?<br>3. How would you interpret results if sputum and stool didn't agree?<br>4. How do you think patients will perceive this method?<br>5. How do you think using stool samples for diagnosing DR-TB will affect perceptions about DR-TB and its transmission?<br>6. How do you think this diagnositc method would affect transmission of TB in the clinic?	

Observations:
