# Supplementary material for: Healthcare providers’ knowledge, attitudes, and perceptions from using targeted sequencing to diagnose and manage drug-resistant tuberculosis (DR-TB) in Eswatini
Source: PLOS Glob Public Health. 2025 Jun 12;5(6):e0004718. doi: 10.1371/journal.pgph.0004718 (PMC12161584; doi:10.1371/journal.pgph.0004718)
Supplement: Attachment — Submitted filename: Response_to_reviewers2.docx [file pgph.0004718.s006.docx]

PGPH-D-24-02931

Healthcare providers’ knowledge, attitudes, and perceptions from using targeted sequencing to diagnose and manage drug-resistant Tuberculosis (DR-TB) in Eswatini

Plos Global Public Health

Dear Dr. Steve Zimmerman,

We sincerely appreciate the reviewer’s insightful comments and the opportunity to revise our manuscript. We have addressed all concerns and believe the revisions significantly improve the paper. Below, we provide detailed responses to each comment.

|  | Comments | Authors’ responses |
| --- | --- | --- |
| **#** | **Introduction** | |
| 1 | You mentioned that in some high-income countries and LMICs, WGS has been implemented for diagnosis of DR-TB. Are there specific cases where targeted sequencing (TS) has been implemented for DR-TB diagnosis? If so, can you provide examples? | Updated in the introduction line 96 to include more information on the specifics of TS implementation for DR-TB diagnosis. |
| 2 | Can you provide a summary of the key challenges and facilitators of implementing targeted sequencing in LMICs specifically? (contextualize the feasibility and scalability of the technology in resource-limited settings). | Considerations for implementing sequencing in LMICs specifically, added to paragraph 2, line 82: “However, stakeholders agreed that there are specific considerations for implementing this technology in LMICs…” |
| 3 | Also, it would be good to highlight the perception or acceptability of WGS in the countries where it has been implemented, if the studies you cited explored these aspects. | Added to paragraph 2 line 79:  “The acceptability of WGS for DR-TB is generally high among clinicians, public health professionals, and policymakers in HICs and LMICs…” |
| 4 | Was TS implemented in Eswatini in 2022 across the country? Or was it piloted in several sites first? Can you please add this information in the introduction? | The details on the implementation in Eswatini have been added to the introduction starting on line 108: “In 2019, with support from the German Ministry of Health, Research Center in Borstel, and Baylor Foundation, Eswatini’s National TB Control Program (NTCP) developed a protocol for implementing TS…” |
|  | **Methods** | |
| 5 | It is good that you give background information about the TB burden and diagnosis in Eswatini. Can you add more information about the implementation of TS by the National TB Control Program? Were doctors and nurses provided with formal training? What preparatory steps were taken to facilitate the implementation of TS in Eswatini? | Details of the preparation to implement TS in Eswatini are now include in the “context” section, starting on line 203: “To address this DR-TB diagnostic gap, in 2019, Eswatini’s NTCP integrated TS into the diagnostic algorithm for DR-TB…” |
| 6 | Can you clarify which levels of the healthcare system were covered in this study? How were the TS results integrated into clinical decision-making, and how did they influence the treatment plans for DR-TB patients? | We spoke with nurses and doctors and NTCP officials. We did not speak with government officials or lab personnel.  When TS results were available, they were used by doctors to inform clinical decision making about which antibiotics to prescribe. Recommendations to treating physicians were made in collaboration with a Clinical Advisory Committee composed of experts in DR-TB. |
| 7 | Can you provide clear inclusion/exclusion criteria for participant selection (like years of experiences, specialty, etc.)? I noticed that in some regions you recruited more participants, was there any rationale behind it? | We clarified the inclusion criteria starting in line 252: “Physicians and nurses working with DR-TB patients at any of the 14 DR-TB sites...”  The number of physicians contacted is included on line 263.  The results of sampling and recruitment has been updated in the sociodemographic section starting on line 323. We clarified that the Hhohho and Shiselweni regions are overrepresented in the study because there are more DR-TB sites in these regions. |
| 8 | Are you aware of the Consolidated criteria for reporting qualitative research (COREQ), which is a commonly used checklist for reporting qualitative studies. Did the reporting of results follow the COREQ checklist or any other established guidelines for qualitative research reporting? | A statement about the COREQ checklist was added to the methods section on line 153: “This study followed the COREQ checklist for reporting qualitative research.”  The checklist is now included as Supplementary material 1. |
|  | **Results** | |
| 9 | What about participants’ knowledge of TS specifically? Did they have knowledge about TS prior to the implementation? Was any training provided to improve their knowledge about TS or TB diagnosis in general? | We added more information about the providers’ knowledge of TS starting in line 480. |
| 10 | The organization of the main results can be improved for clarity. For example, does Table 2 covers all the main themes in the results section or only the “Healthcare providers perceive challenges in diagnosing DR-TB” section?  Also, in the section "Healthcare providers perceive challenges in diagnosing DR-TB," themes are presented in a table with participant quotes but without further explanation.  Since it is likely that multiple participants commented on each theme, summarizing the overall patterns or trends observed across participants would provide a clearer and more informative synthesis of the findings. | The results section has been updated to include more quotations with explanations in the text.  Table 2 covers main themes discussed throughout the results section, not just in the “Healthcare providers perceive challenges in diagnosing DR-TB” section. To clarify the connection between the themes, sociodemographic data and the synthesized results, we added a **“themes”** section at the beginning of the results. Table 2, which introduces themes and illustrative quotes to be discussed throughout the results, has been moved to the themes section.  Additionally, Figure 3 has been updated to include the synthesized findings on sputum-based TS. |
|  | **Discussion** |  |
| 12 | In countries where TS or WGS was implemented, were there specific preparatory steps taken before implementation? If so, what lessons can Eswatini learn from these experiences to enhance the successful adoption and integration of TS into its healthcare system? | Lessons on implementation challenges from Namibia were added to the discussion, starting on line 786. |
| 13 | Since cost was discussed, can you provide more details on who covers the cost of testing in the countries you cited? Are TS or WGS tests funded by governments, international donors, or private institutions? How is the cost of TS managed in Eswatini, and what funding mechanisms are currently in place to support its implementation? | The funding for sequencing in Eswatini has been added to the introduction and context sections (see response to comment 4).  A discussion of funding sources has been added to starting on line 756: “Moreover, most sequencing in LMICs is funded by governments…” |

We sincerely hope that our revisions and responses have addressed all concerns. We believe the manuscript is now stronger and more comprehensive. Thank you again for your valuable feedback.

Sincerely,

Maia Madison
